# Neurophysiological and emotional influences on team communication and metacognitive cyber situational awareness during a cyber engineering exercise

**DOI:** 10.3389/fnhum.2022.1092056

**Published:** 2023-01-05

**Authors:** Torvald F. Ask, Benjamin J. Knox, Ricardo G. Lugo, Ivar Helgetun, Stefan Sütterlin

**Affiliations:** ^1^Department of Information Security and Communication Technology, Norwegian University of Science and Technology, Gjøvik, Norway; ^2^Faculty for Health, Welfare and Organization, Østfold University College, Halden, Norway; ^3^Norwegian Armed Forces Cyber Defense, Lillehammer, Norway; ^4^Norwegian Defense University College, Cyber Academy, Lillehammer, Norway; ^5^Faculty of Computer Science, Albstadt-Sigmaringen University, Sigmaringen, Germany; ^6^Centre for Digital Forensics and Cyber Security, Tallinn University of Technology, Tallinn, Estonia

**Keywords:** vagal tone, cognitive control, cyber operations, neuroergonomics, metacognition, cyber situational awareness, emotion, cyber team communication

## Abstract

**Background:** Cyber operations unfold at superhuman speeds where cyber defense decisions are based on human-to-human communication aiming to achieve a shared cyber situational awareness. The recently proposed Orient, Locate, Bridge (OLB) model suggests a three-phase metacognitive approach for successful communication of cyber situational awareness for good cyber defense decision-making. Successful OLB execution implies applying cognitive control to coordinate self-referential and externally directed cognitive processes. In the brain, this is dependent on the frontoparietal control network and its connectivity to the default mode network. Emotional reactions may increase default mode network activity and reduce attention allocation to analytical processes resulting in sub-optimal decision-making. Vagal tone is an indicator of activity in the dorsolateral prefrontal node of the frontoparietal control network and is associated with functional connectivity between the frontoparietal control network and the default mode network.

**Aim:** The aim of the present study was to assess whether indicators of neural activity relevant to the processes outlined by the OLB model were related to outcomes hypothesized by the model.

**Methods:** Cyber cadets (*N* = 36) enrolled in a 3-day cyber engineering exercise organized by the Norwegian Defense Cyber Academy participated in the study. Differences in prospective metacognitive judgments of cyber situational awareness, communication demands, and mood were compared between cyber cadets with high and low vagal tone. Vagal tone was measured at rest prior to the exercise. Affective states, communication demands, cyber situational awareness, and metacognitive accuracy were measured on each day of the exercise.

**Results:** We found that cyber cadets with higher vagal tone had better metacognitive judgments of cyber situational awareness, imposed fewer communication demands on their teams, and had more neutral moods compared to cyber cadets with lower vagal tone.

**Conclusion:** These findings provide neuroergonomic support for the OLB model and suggest that it may be useful in education and training. Future studies should assess the effect of OLB-ing as an intervention on communication and performance.

## 1 Introduction

Cyber operations unfold at superhuman speeds, which pose high demands on human cyber operators. Due to the growing global network coverage and increasing interconnectedness between cyber and physical domains, cyber operations are conducted in a complex socio-technical system consisting of diverse human-machine and human-human interactions. Performance in this socio-technical system is influenced by several factors across multiple contexts including unique challenges spanning cyber, physical, cognitive, and social domains (Jøsok et al., 2016, 2017; Agyepong et al., 2019). The resulting working-environment poses a complex selective pressure requiring a seemingly unique but currently understudied competency profile (Jøsok et al., 2017, 2019; Knox et al., 2017, 2018; Lugo and Sütterlin, 2018).

**Figure 1:**
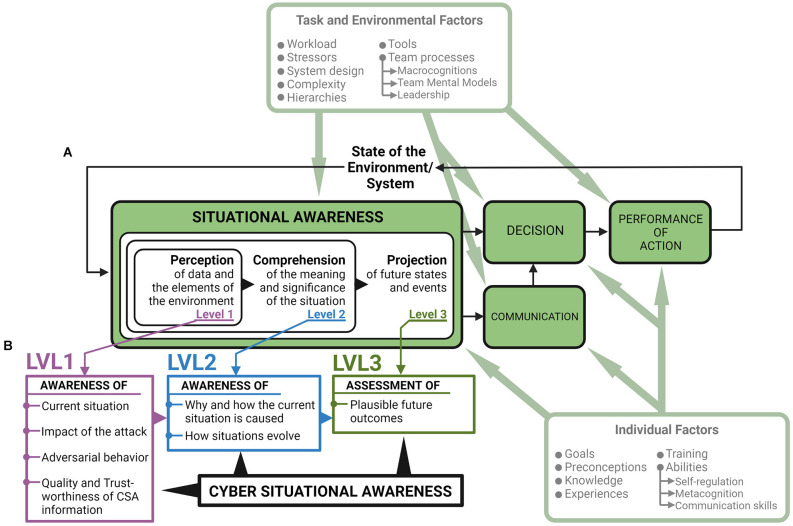
Situational Awareness (SA) model with suggested requirements for achieving Cyber Situational Awareness (CSA). **(A)** The SA model (Endsley, 1995). Communication has been added to the model due to its role in Security Operation Center (SOC) team decision-making (Knox et al., 2018; Ask et al., 2021a). **(B)** Seven requirements for achieving CSA during cyber threat situations (Barford et al., 2009). CSA generation, CSA sharing, and subsequent decision-making is affected by individual factors such as metacognition, self-regulation, and communication skills (Jøsok et al., 2016, 2019; Knox et al., 2017, 2018; Knox et al., 2019; Sütterlin et al., 2022) and task and environmental factors such as team-processes including macrocognitions, team mental models, and leadership (Jøsok et al., 2017; Lugo et al., 2017a; Ask et al., 2021a, b). Modified from Lankton (2007).

Organizations source their cyber operations to Security Operation Centers (SOCs) consisting of teams and organizational units that work around the clock to detect, assess, and respond to cyber threats. SOCs are usually hierarchically organized where analyst-level responsibilities such as detecting, investigating, and reporting on cyber threats are assigned to technical personnel (cyber operators), while decision-making responsibilities are assigned to other individuals higher up in the SOC hierarchy (Staheli et al., 2016). Thus, cyber operators are responsible for establishing situational awareness (SA) during cyber threat situations and communicating their SA to decision-makers. According to the SA model (Endsley, 1995), establishing SA for decision-making in a socio-technical system is achieved in three levels (Figure 1A), where all levels must be achieved in order to have full SA. SA Level 1 entails perceiving the elements of the situation, SA Level 2 entails comprehending the relationship between these elements, and SA Level 3 entails using the comprehension to predict possible future situational states (Endsley, 1995).

**Figure 2:**
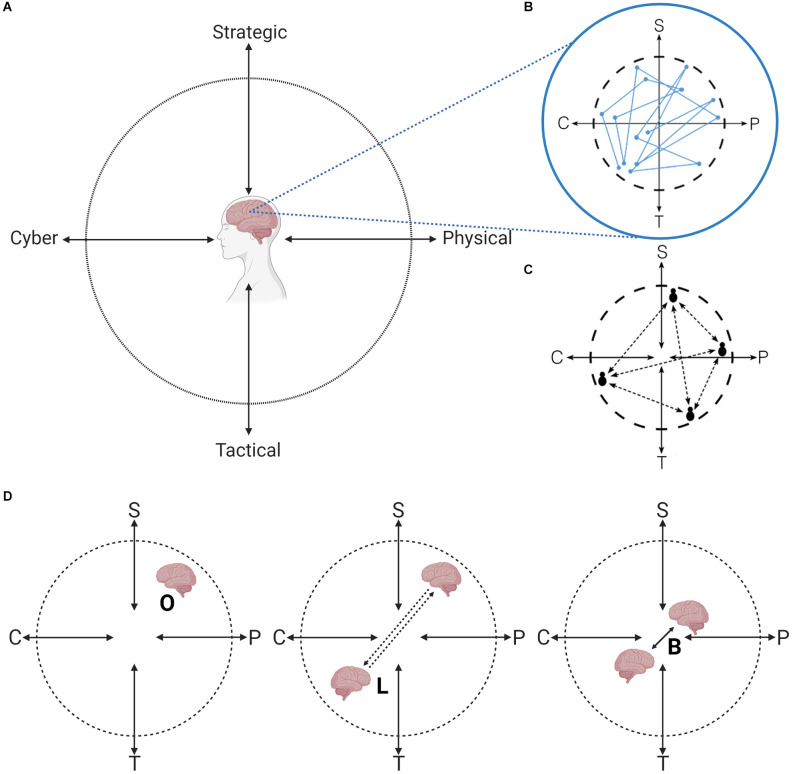
The Hybrid Space (HS) framework and the OLB model. **(A)** The Hybrid Space (HS; Jøsok et al., 2016). **(B)** Self-location and movement in the HS require metacognition and self-regulation (Knox et al., 2017, 2019; Jøsok et al., 2019). **(C)** Communication between individuals located in different quadrants of the HS. **(D)** The OLB model. S, Strategic; P, Physical; T, Tactical; C, Cyber; OLB, Orient, Locate, Bridge. Figure adapted from Jøsok et al. (2016) and Ask et al. (2021b); Ask et al. (2022). Created with BioRender.com.

Seven requirements for achieving cyber SA (CSA) for decision-making during cyber threat situations have been proposed (Barford et al., 2009). These requirements can be arranged under the SA model (Figure 1B) where the establishment of SA Level 1 starts with having perceived indicators of compromise (Barford et al., 2009). Establishing shared CSA during a cyber threat situation depends on both technical expertise and socio-cognitive abilities (Franke and Brynielsson, 2014; Jøsok et al., 2016, 2019). The outcome of cyber defense decision-making is based on how well the cyber operators can communicate their CSA to decision-makers that are often less technically competent (Knox et al., 2018; Ask et al., 2021a). Cyber operators must therefore be capable of flexibly transitioning between cyber-oriented analytical processes and socially oriented processes such as adjusting communication to the needs of the recipient. This makes communication for shared CSA a dynamic and challenging process where the same complex information is communicated in different ways depending on the recipient’s background (Ahrend et al., 2016; Staheli et al., 2016). The Hybrid Space framework (Figure 2A) was developed to illustrate the interconnectedness between the cyber and physical (cyber-physical) domains, and the tension between strategic and tactical goals in decision-making and action, thus outlining the cognitive landscape that cyber operators must navigate (Jøsok et al., 2016).

Transitioning between quadrants in the Hybrid Space to relay technical information to non-technical individuals, will in theory require the cyber operator to switch between mindsets (Jøsok et al., 2016; Knox et al., 2018). Doing this effectively depends on the cyber operator’s ability to monitor and regulate their cognitions. Metacognition is the ability to direct attention internally to observe one’s own cognitions, emotions, and behaviors, and assess if they align with goals, and consciously regulate them if needed (Flavell, 1979; Efklides, 2008). Metacognition is required for establishing accurate SA (Endsley, 2020). Previous studies on cyber cadets have shown that self-location and movements in the Hybrid Space is predicted by metacognition and self-regulation (Figure 2B; Knox et al., 2017, 2019; Jøsok et al., 2019) and team communication measurements (Lugo et al., 2017a). When individuals are processing information in different domains, their cognitive focus is in different quadrants of the Hybrid Space. Communicating across quadrants of the Hybrid Space (Figure 2C) requires constant re-adjustment of communication flow and message content (Jøsok et al., 2017; Knox et al., 2018).

Recent reviews suggest that there is a lack of research on individual- and team-level SOC team communication and performance indicators (Agyepong et al., 2019; Ask et al., 2021a). The Orient, Locate, Bridge (OLB) model (Figure 2D; Knox et al., 2018) was developed based on the Hybrid Space framework to serve as a metacognitive tool supporting communication and sharing of CSA between individuals located in different quadrants of the Hybrid Space. The OLB model is a three-phase model where each phase builds on the previous phase to facilitate efficient communication (Knox et al., 2018). In the orienting phase, the cyber operator applies metacognition to self-locate in the Hybrid Space to get a grasp of their current mindset (e.g., where their focus is, if they are stressed, etc.) and their CSA. In the locating phase, the cyber operator applies perspective taking to understand the specific information and communication needs of the recipient based on their location in the Hybrid Space. In the bridging phase, the cyber operator uses the insights from the orienting and locating phases as a guide for adapting communication style and content. This last phase ensures that communication can be grounded and CSA can be shared and calibrated between the cyber operator and the communication partner.

In more general terms, OLB-ing can be understood as a stepwise cognitive control process involving the deliberate (endogenously controlled) and flexible transition between attention to internal and self-referential states (e.g., Hybrid Space location, stress levels) and externally oriented cognitive processes. Cognitive control is the goal-directed coordination of task-relevant cognitive processes while inhibiting task-irrelevant automatic processes (Friedman and Robbins, 2022). In the brain, goal-directed cognitive processes are organized by a network of brain areas called the frontoparietal control network (FPN; Duncan, 2010; Menon and D’Esposito, 2022). This includes integrating attention to information from the external-present task environment and attention to internal-future goal-representations to coordinate externally directed cognitive processes and behaviors towards goal attainment (Nee and D’Esposito, 2016; Nee, 2021). On the other hand, attention to internal and self-referential information such as thoughts or the intensity and significance of one’s emotional state is processed and maintained by the default mode network (DMN; Raichle et al., 2001; Raichle, 2015).

Both the FPN and DMN have anatomical hubs in the prefrontal cortex (PFC; Raichle, 2015; Menon and D’Esposito, 2022). The dorsolateral PFC (DLPFC) is one of the main hubs in the FPN (Menon and D’Esposito, 2022), while medial PFC structures (MPFC) constitute one of the main hubs of the DMN (Raichle, 2015). Activity in the FPN and DMN is often anticorrelated (Raichle et al., 2001; Fox et al., 2005; Chang and Glover, 2009), and DLPFC and MPFC activity is often anti-correlated during FPN-related tasks (Chen et al., 2013; Liston et al., 2014).

Both the DLPFC and MPFC are involved in metacognitive processes (Fleur et al., 2021). The DLPFC is specifically involved in metacognitive decision-making, while both DLPFC and MPFC are involved in prospective metacognitive judgments (Vaccaro and Fleming, 2018; Fleur et al., 2021). Cognitive processes can go from being metacognitively controlled (Shimamura, 2008; Friedman and Robbins, 2022) to reactive (stimulus-driven) when individuals are subjected to stress or under emotional influence (Baek and Falk, 2018; Poth, 2021). Previous studies on cyber operators identified several emotional processes that may have differing effects on teamwork and communication (Lugo et al., 2016, 2017b, 2021; Ask et al., 2021b). Emotions can be interpreted according to intensity (arousal) and whether they are positive, negative, or neutral (valence; or mood), and are processed differently by DMN and FPN structures (Golkar et al., 2012; Terasawa et al., 2013; Fujimoto et al., 2021; Nejati et al., 2021). Stress reduces connectivity between the DMN and FPN (Chand et al., 2020), suggesting ways for how environmental pressures can disturb the flexible transition between self-referential and analytical processing.

From a neuroergonomics perspective, when faced with a challenging environment, the brain will find something akin to “the path of least resistance” to optimal performance (Botvinick, 2007; Wickens et al., 2015; Hagura et al., 2017; Khalil et al., 2019). This means initiating the cognitive processes and behaviors that are the least taxing to apply in order to reach a goal, given the environmental demands. If resulting in goal-attainment, they become part of a strategy for reaching the same goals under similar circumstances. The precision, combination, and order of the cognitions and behaviors, or trying new strategies to compare efficiency with older successful ones, are deliberately or unconsciously adjusted with experience *via* metacognitive and cognitive control processes (Flavell, 1979; Efklides, 2008; Khalil et al., 2019). Because the cybersecurity working-environment places such a heavy cognitive load on cyber operators (Jøsok et al., 2016; Agyepong et al., 2019), strategies for improved communication must not only be successful, but they must also be neuroergonomic to be sustainable. If the processes outlined by the OLB model are both successful and neuroergonomic in a cybersecurity working-environment, then they should be selected through evolutionary processes by the individuals working in those environments. If so, correlates of the neurocognitive processes underlying OLB-ing should be related to the outcomes predicted by the model (Knox et al., 2018).

Expert cyber incident response teams impose less communication demands on their teams compared to novices (Buchler et al., 2016; Lugo et al., 2017a). Because experts have spent more time in cybersecurity working-environments, they have also had more time to go through evolutionary cycles for selecting neuroergonomic approaches to make communication more efficient. If the OLB model is neuroergonomic, the discrepancy in imposed communication demands between novices and experts may suggest that expert teams have a higher conscious or unconscious adoption rate of OLB-related cognitive processes for communication.

The main aim of this study is to assess some of the neurocognitive assumptions of the OLB model (Knox et al., 2018) to begin validating its potential as a neuroergonomic approach for CSA communication in cyber threat situations. This is done using peripheral proxies for DLPFC activity and FPN-DMN connectivity, and measurements of CSA, metacognition, and team communication. Prefrontally modulated vagal tone, quantified as vagally mediated heart rate variability (vmHRV), represents the beat to beat variations in heart rate that are influenced by the vagus nerve and modulated by the PFC (Appelhans and Luecken, 2006; Thayer et al., 2012). Vagal tone is an indicator of DLPFC activity (Brunoni et al., 2013a; Nikolin et al., 2017) and functional connectivity between the FPN and the DMN at rest (Chand et al., 2020). Associations have been found between metacognition and vagal tone in non-cyber tasks (Meessen et al., 2018). Vagal tone may therefore serve as a potential marker for the FPN-DMN interactions relevant for OLB-ing during cyber operations. Thus, we hypothesize that individuals with higher vagal tone have higher metacognitive accuracy and impose lower communication demands on their teams than individuals with lower vagal tone (hypothesis 1: H_1_).

The processing of emotional stimuli may influence cyber team performance (Lugo et al., 2016, 2017b, 2021; Ask et al., 2021b) for example by diverting attention away from externally directed and endogenously controlled cognitive processing and more towards stimulus-driven external (Poth, 2021) or internally directed self-referential processing (Baek and Falk, 2018). The DLPFC is involved in the self-report of valence (Nejati et al., 2021) and can be distinguished from other prefrontal structures based on this function (Terasawa et al., 2013; Fujimoto et al., 2021; Nejati et al., 2021). Higher vagal tone is associated with stress resilience (Hildebrandt et al., 2016) and endogenous control over attention during emotional distractors (Geisler and Kubiak, 2009; Park et al., 2012, 2013) known to elicit DMN processing (Winston et al., 2003; Zhou et al., 2020). Thus, we hypothesize that individuals with higher vagal tone, which reflects DLPFC function, will have different self-reported mood ratings than individuals with lower vagal tone (H_2_).

Metacognition is required for establishing accurate SA (Endsley, 2020). The Hybrid Space framework and the OLB model suggest that metacognition is required for efficient communication of CSA with other individuals in the Hybrid Space (Jøsok et al., 2016; Knox et al., 2018). Metacognitive accuracy, how correctly an individual is in evaluating their own performance, can manifest as correctly judging performance as bad or good. Because both establishing SA and sharing CSA through communication is reliant on metacognition (Jøsok et al., 2016; Knox et al., 2018; Endsley, 2020), we hypothesize that individuals with more correct CSA ratings have higher metacognitive accuracy than individuals with less correct CSA ratings (H_3_).

## 2 Methods

### 2.1 Participants and setting

**Figure 3:**
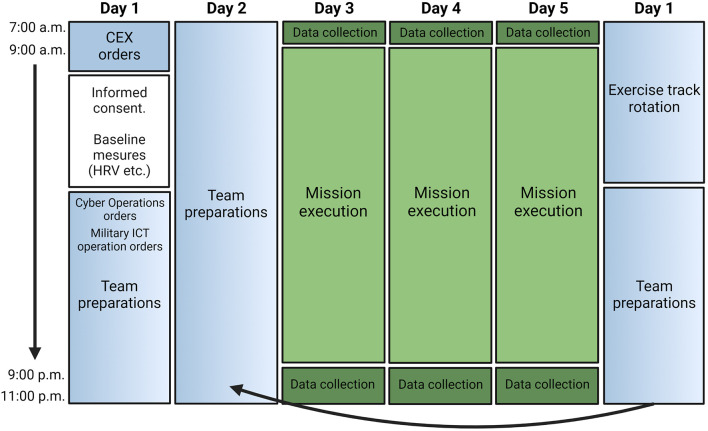
Overview of the study and the cyber engineering exercise. CEX, Cyber engineering exercise; HRV, Heart rate variability.

Cyber cadets (*N* = 36) that participated in the Norwegian Defense University College, Cyber Academy (NDCA) annual Cyber Engineering Exercise (CEX) were recruited for the study. The CEX is conducted during the fifth semester for graduating students at the NDCA. By this stage in their bachelor degree education, they have chosen and begun their specialized training. The specializations are military Information Communication Technology (ICT) systems and Cyber Operations. The specialization split was eleven (11) cadets pursuing Cyber Operations and the remaining twenty-five (25) military ICT. The CEX is intended to provide cyber cadets with a deeper understanding and appreciation for the breadth of a cyber engineer’s profession and tasks in a military operative context. In particular, they develop more advanced technical skills in the domain of cyber operations and gain insight into how incidents occurring in the military cyber domain may influence and be influenced by operations in other military and non-military domains. The cadets learn how to make good judgments, give honest recommendations through clear communication, and make good decisions that result in the effective use of cyber tools and technologies to achieve operational goals. The CEX was divided into two independent operations: military ICT Operations and Cyber Operations. Both operations lasted for 5 days (see Figure 3 for an overview of the CEX and study).

For the CEX, the cadets were divided into two platoons, with each platoon consisting of three teams. One platoon participated in military ICT Operations for 5 days, while the other platoon participated in Cyber Operations for 5 days. Each team consisted of six individuals that were composed of a mix of military ICT and Cyber Operations cadets. The first 2 days involved orders, preparation, and training. This was followed by 3 days of mission execution. On day 6, there was a rotation where the platoons switched operations so that the platoon that started out in the military ICT operations track switched to the Cyber Operations track, and *vice versa*. Participants per rotation (*n* = 16).

The present study took measurements when each platoon was undertaking the Cyber Operations track of the CEX. The defensive Cyber Operations involved scenario-based investigations of a network intrusion where the cadets experienced technical and operational uncertainty and complexity related to exploitation of their military cyber domain. After the initial preparation and training phase, the cadets deployed to a notional area of operations. For the CEX, this took the form of teams being assigned separate rooms, where the cadets deployed network sensor capabilities into their infrastructure and began targeted network surveillance based on their operational assessment and plans. The scenario developed allowing cadets to conduct different analytical tasks and investigate specific types and instances of network traffic. The cadets advanced through the exercise by solving these analytical and investigative missions. Each day the exercise would begin at 8:00 a.m. and end at 10:00 p.m., with the level of intensity (operational uncertainty and technical complexity) imposed upon the cadets gradually increasing each day. There were organized regular breaks for eating three times per day, once in the morning, once around noon, and once in the evening, where all the cadets participating in the Cyber Operations track could eat simultaneously. If any of the teams operated in shifts, this was organized within the teams, but usually meant that someone would bring with them food from the cafeteria to the individual(s) that did not join the common breaks.

Data from four participants were excluded from the analysis. Data from one participant were excluded for only providing baseline HRV data and not filling out daily questionnaires. A second participant was excluded due to measurement error during the recording of inter-beat intervals. Lastly, two participants were excluded due to not filling out relevant questionnaires for most of the exercise.

### 2.2 Materials and procedure

On the first day of the study, 2 days before the start of the CEX all participants answered a battery of questionnaires followed by recording of cardiac activity for quantification of vmHRV. Affective state, performance rating, team, and CSA measurements were collected on each day of the exercise. On the morning of each day of the exercise (approx. 7:30 a.m.), participants answered questionnaires pertaining to their affective state and judgments about how well they think they would perform. At the end of each day (approx. 9:00 p.m.), participants answered questionnaires pertaining to judgments about how well they thought they had performed, team-workload demands, and CSA.

#### 2.2.1 Vagally mediated heart rate variability

Cardiac activity was recorded at rest for 7 min 2 days prior to the start of the exercise using the Alive Software (Alive^TM^ by Somatic Vision, Inc., Encinitas, CA, United States) biofeedback system. Alive measures heart rate through photoplethysmography. The recordings were conducted one at a time in a separate room that was secluded from other activities. Participants were seated in comfortable chairs. Three finger sensors were placed on the participant’s non-dominant hand, after which they were told to rest for some minutes by themselves. After giving the instructions, the researcher left the room for the entirety of the 7-min recording period.

Five minutes in the middle of the recordings were used for quantification of vmHR. Inter-beat intervals were extracted *via* R-peak detection and HRV was analyzed using ARTiiFACT software (Kaufmann et al., 2011). Artifacts were detected and corrected according to established methods (Berntson and Stowell, 1998). The high frequency component of HRV (HFHRV: 0.15–0.40 Hz, ms^2^) and the time-domain measure of HRV, root-mean-squares of successive NN differences (RMSSD), were extracted following recommendations by the Task Force of the European Society of Cardiology and the North American Society of Pacing and Electrophysiology (1996). Both indices are commonly used as indicators of vagal tone. We were mainly interested in HFHRV as RMSSD is suggested to also be influenced by sympathetic input (Berntson et al., 2005; Williams et al., 2019) and evidence relating transcranial stimulation over DLPFC to prefrontally modulated vagal tone used HFHRV as an indicator (Brunoni et al., 2013a; Nikolin et al., 2017). HFHRV and RMSSD are usually highly correlated (Goedhart et al., 2007), thus we included RMSSD in the initial analysis to check for this correlation as an indicator of vmHRV index quality.

#### 2.2.2 Self-assessment manikin

The self-assessment manikin (SAM) is a non-verbal assessment of affective states (Bradley and Lang, 1994). It consists of three pictorial items: Valence (mood; ranging from very negative to very positive), arousal (activation; ranging from very low to very high), and dominance (control; ranging from very low to very high) that are each measured on a 9-point scale, where participants indicate what they feel in the moment for each item. The SAM was administered at the beginning of each day during the exercise. The mean for mood, activation, and control scores were computed for each individual for all 3 days.

#### 2.2.3 Judgment of performance

A prospective judgment of performance (JOP) questionnaire was used to assess the participants’ prospective estimations of how well they would perform. The JOP questionnaire is used to assess how confident participants are about their future performance (e.g., Sütterlin et al., 2022). The questionnaire consisted of six items that were handed out at the beginning and at the end of each day, and included questions such as “How well do you think you will do?”, and “How well will my team do?”. The prospective JOP questionnaires were handed out at the beginning of each day. On each item, participants indicated their performance on a scale ranging from 0% to 100%.

For this study, daily perspective JOP at the individual and team level were z-transformed before averaging to generate prospective self-assessment (JOP) and team assessment (JOP team) JOP scores.

#### 2.2.4 Team workload questionnaire

Establishing CSA is a team effort (McNeese et al., 2011; Champion et al., 2012; Jøsok et al., 2017). The team workload questionnaire (Sellers et al., 2014) was administered at the end of each day to assess how participants experienced workload demands on team tasks during the exercise. The items are scored on an 11-point Likert scale ranging from very low to very high. High scores indicate higher levels of subjective workload. The team workload questionnaire consists of six subscales divided into two dimensions, the Teamwork component (communication, coordination, team performance monitoring) and Task-Team component (time-share, team emotion, team support). Average scores for all 3 days were generated based on these subscales. The team workload questionnaire shows good reliability (Sellers et al., 2014). Reliability was also good in the present study (Cronbach’s *α* = 0.839). The perceived team success item from the NASA Task Load Index assesses retrospective confidence judgments of team performance (Hart and Staveland, 1988) and was also included in the daily team workload questionnaire.

#### 2.2.5 Cyber situational awareness questionnaire for analysts

To assess CSA among participants, the CSA questionnaire for analysts (Lif et al., 2017) was administered at the end of each day. The questionnaire consists of a combination of qualitative and quantitative questions. Among the questions included in the CSA questionnaire, the following four were used for the purpose of study 1: “Where in the Kill Chain is attack 1?” (Kill chain), “How critical is the system?” (System critical), “How Severe is the Attack?” (Attack severity), “How urgent is it to take action?” (Action urgency).

For all CSA items, participants had to indicate which estimate they thought was correct on a Likert scale from 1 to 7. For the Kill chain item, participants had to indicate on a Kill chain flow chart where the attack was (seven options). Their answers were converted to a score from 1 to 7 depending on where in the kill chain they indicated that the attack was, with 7 corresponding to “Action on objectives”, which is the last step in the kill chain.

Participants were instructed to leave items they did not know what to answer blank, but to write their participant ID on the front page, in which case those items were coded as 0 (thus making CSA items range from 0 to 7). Responses were coded as missing if the entire form was left empty. Reliability for CSA items was good (Cronbach’s *α* = 0.771).

The correct answer for the kill chain item was 7. The correct answer for attack severity was 7. The correct answer for action urgency was 7. The correct answers for system critical was 6. CSA scores for the participants were generated by scoring correct assessments on the questionnaires for each day as 1 and erroneous assessments as 0. The scores for each day were z-transformed before averaging to generate CSA scores. Kill chain estimations could in theory be inferred from exercise instructions thus being too easy to tax metacognitive abilities. Metacognitive estimations for easy tasks are less subject to bias than for harder tasks (Fleur et al., 2021). Accounting for this possible confounder, a second CSA score was generated through the same steps as for the initial CSA variable except kill chain scores were excluded, resulting in a CSA2 variable.

The same procedure was done at the team level, where team CSA scores were generated based on the averaged correct CSA estimations for the entire team, and a team CSA2 variable was generated by excluding kill chain scores.

Due to the structure of the exercise, participants would only be able to make informed judgments on the attack severity item on days 4 and 5, while informed judgments on the kill chain, how critical the system was, and action urgency were possible on all 3 days. There was a very low number of correct CSA answers on day one which was likely due to participants spending time on sensor deployment and only starting to establish CSA during the tail end of the day.

#### 2.2.6 Metacognitive accuracy

The individual and team CSA scores for each day, and the personal and team JOP scores for each day were used to generate the metacognitive accuracy (MCA) scores. CSA scores were range converted to a 0–100 scale. MCA scores were calculated as a deviation score using the approach described by Meessen et al. (2018). Briefly, scores were generated by squaring the product from subtracting the daily JOP scores (ranging from 0 to 100) for each day from the daily CSA scores (ranging from 0 to 100) for each day. This was followed by dividing by 100, using the following formula:


$$\text{M}CA=\frac{{\left(Daily\;CSA\;score-daily\text{ J}OP\right)}^{2}}{100}$$

Because JOP scores are subtracted from the accuracy scores, CSA performance that matches performance estimations will give a score of zero, while performance estimations that are below or above CSA performance will give a score that deviates from zero. Squaring the product returns an equal positive value for all negative and positive equivalent deviations from zero. Thus, a low MCA score indicates high metacognitive accuracy, and a high MCA score indicates low metacognitive accuracy regardless of inaccuracy resulting from overconfidence or underconfidence. At the team level, a high metacognitive accuracy means having high accuracy when judging team-level CSA.

The z-transformed MCA scores for each day were averaged to generate two sets of MCA variables, MCA and team MCA. While the DLPFC is needed for making prospective metacognitive judgments about performance (Vaccaro and Fleming, 2018), metacognition for team processes is suggested to rely on different neural systems than those for individual metacognitions (Shea et al., 2014). Following the rationale for generating the CSA variables, two separate MCA variables were generated for each set: MCA and team MCA, and MCA2, and team MCA2, where the MCA2 variables excluded Kill chain scores.

### 2.3 Statistical analysis

Descriptive statistics were generated for all variables and presented in tables as mean, standard deviation (SD), minimum (min), and maximum (max) values for continuous and numerical variables, and frequencies and percentages (%) for ordinal variables.

Inspecting box-and-whisker plots of variables identified one outlier (value > 1.5 times the interquartile range above the upper quartile) for HFHRV. After re-inspection of inter-beat interval recording and artifact analysis for the HFHRV outlier, it was concluded that measurement error was unlikely, thus, HFHRV was log-transformed to pull in the outlier. Follow-up inspecting box-and-whisker plots confirmed that the log-transformed variable no longer contained extreme values. All subsequent analyses were performed on the log-transformed HFHRV variable.

For the purpose of the present study, we were mainly interested in the communication demand item of the team workload questionnaire due to reported differences between expert and novice teams (Buchler et al., 2016; Lugo et al., 2017a), and because the OLB model aims to reduce communication demands by making communication more efficient (Knox et al., 2018). Because establishing CSA is a team effort (McNeese et al., 2011; Champion et al., 2012; Jøsok et al., 2017), all team workload questionnaire items were included in the analysis to assess the relationship between team workloads and team-level CSA and MCA. All variables were z-transformed for analysis. Shapiro-Wilk test of normality and confirmatory visual inspection of bar-graph distribution plots revealed that many of the variables were not normally distributed. Subsequent correlational analyses were parametric for relationships between normally distributed variables (SAM, HFHRV, MCA2, team MCA, team performance monitoring, team support demand), and nonparametric for all other relationships including between normally and not normally distributed variables.

Pearson and Spearman correlation analyses (2-tailed) was performed simultaneously for all variables and results were presented in a heat map as Spearman correlation coefficients (ρ) for nonparametric associations and Pearson’s correlation coefficients (*r*) for parametric associations. RMSSD was included in the correlation analysis to check for associations with HFHRV but was not included in the heat map. Separate linear regression analyses were performed for significant correlations. All regressions were checked for violation of assumptions regarding homoscedasticity, normality, linearity, and multicollinearity.

#### 2.3.1 Analysis of group differences

The differences between high and low HFHRV groups were assessed using Pillai’s MANOVA and ANOVA for parametric comparisons and Kruskal-Wallis H tests for nonparametric comparisons. Results for the Pillai test were reported as Pillai’s Trace (Trace_Pillai_), approximate *F*(degrees of freedom 1, degrees of freedom 2; *F*_(df1, df2)_), and *p*-values. Results for ANOVA were reported as *F* statistic(df), *p*-values, and effect size. Kruskal-Wallis *H* test was reported as *H* statistic(df), *p*-values, and effect size.

Effect size (η^2^) for the Kruskal-Wallis H test was calculated as (H−k + df)/(n−k); where H was the Kruskal-Wallis statistic, *k* was the number of groups, and *n* was the total number of observations (32). Effect size (ω^2^) for ANOVA was calculated as (sum of squares between − (k − 1) mean square within)/(sum of squares total + mean square within). Dunn’s *post-hoc* test was used to assess significant relationships for non-parametric variables between groups and was reported as z-statistic and Bonferroni adjusted *p*-values (*p_bonf_*). Tukey’s *post-hoc* test was used to assess significant relationships for parametric variables between groups and was reported as mean difference (MD) and *p_bonf_*.

Violation of assumptions for MANOVA analyses were assessed with Box’s M-test for homogeneity and Shapiro-Wilk test for multivariate normality. Violation of assumptions for ANOVA analyses were assessed with Levene’s test for equality of variance and by inspecting Q-Q plots of residuals. There were no violations at any time.

#### 2.3.2 Comparisons between low and high vagal tone groups

A median split was performed on the HFHRV variable to divide the sample into high HFHRV (HFHRV > median) and low HFHRV (HFHRV ≤ median) groups according to whether they had values above or below the median. This method is commonly used in studies aiming to assess vagal tone-related group differences in cognitive performance (Hansen et al., 2003, 2009; Pu et al., 2010; Williams et al., 2017). To test the hypotheses that individuals with higher vagal tone have higher metacognitive accuracy and impose lower communication demands on their teams than individuals with lower vagal tone (H_1_), and that individuals with higher vagal tone have different self-reported mood ratings than individuals with lower vagal tone (H_2_), ANOVAs were performed using vagal tone groups as fixed factors and mood, communication demand, and MCA variables as dependent variables. Metacognition has been suggested to be required for SA (Endsley, 2020) so CSA variables were also included in the analysis.

#### 2.3.3 Comparisons between low and high metacognitive accuracy groups

Both vagal tone and prospective metacognitive judgments are influenced by DLPFC activity (Brunoni et al., 2013a; Nikolin et al., 2017; Vaccaro and Fleming, 2018; Chand et al., 2020). However, vagal tone can be influenced by processes other than DLPFC activity (Task Force of the European Society of Cardiology and the North American Society of Pacing and Electrophysiology, 1996), but prospective metacognitive judgments are dependent on DLPFC activity (Fleur et al., 2021). Thus, a median split was performed on MCA and team MCA to assess whether the vagal tone was different between individuals with high and low MCA and team MCA. Due to low MCA scores meaning high accuracy, individuals below the median had high accuracy, and individuals above the median had low accuracy.

#### 2.3.4 Comparisons of MCA between CSA accuracy groups

In the present study, high metacognitive accuracy (indicated by low MCA scores) could be due to accurately judging good or bad performance (e.g., having 0% correct answers and judging performance at 0%, and having 100% correct answers and judging performance at 100% would both give a score of 0). A median split was performed on the summed total of correct CSA ratings for both days to divide the sample into two groups (CSA accuracy) according to whether they were less accurate or more accurate in their CSA ratings. To test the hypothesis that individuals with higher metacognitive accuracy have more correct CSA ratings than individuals with lower metacognitive accuracy (H_3_), two separate analyses were performed using MCA or MCA2 as a dependent variable and CSA accuracy as the fixed factor. This procedure was repeated for team MCA variables also, where the median split was performed on the summed total of correct team CSA ratings after averaging for the number of team members.

Alpha levels for hypothesis testing were set at the 0.05 level for all analyses. All data were analyzed using JASP version 0.15 (JASP Team, 2021).

## 3 Results

### 3.1 Descriptive statistics

Descriptive statistics for HRV indices, SAM, team workload questionnaire, JOP, CSA, and MCA variables are presented in Table 1.

**Table 1:** Descriptive statistics for HRV, SAM, TWLQ, JOP, CSA, and MCA variables (*N* = 32).

					**High HFHRV**		**Low HFHRV**	
**Variables**	**Mean**	**SD**	**Min**	**Max**	**Mean**	**SD**	**Mean**	**SD**
Mean RR	935.73	174.06	677.63	1,342.760	1,023.570	175.60	847.89	123.78
HFHRV	1,473.190	1,501.760	124.82	5,079.730	2,547.500	1,471.220	398.88	185.10
HFHRV_log	6.75	1.09	4.82	1.62	7.65	0.68	5.86	0.54
RMSSD	60.92	28.38	32.08	136.95	82.05	26.03	39.80	5.87
CSA	0.23	0.17	0.00	0.75	0.26	0.19	0.20	0.14
CSA2	0.18	0.17	0.00	0.66	0.22	0.19	0.14	0.15
Team CSA	0.23	0.67	0.18	0.36	0.24	0.07	0.22	0.05
Team CSA2	0.18	0.84	0.74	0.33	0.21	0.07	0.15	0.08
Z-Transformed variables								
HFHRV	−0.00	1.00	−1.76	1.62	0.81	0.62	−0.81	0.50
RMSSD	0.00	1.00	−1.01	2.67	0.74	0.91	−0.74	0.20
Mood	0.00	1.00	−2.02	1.84	−0.47	0.90	0.47	0.87
Activation	−0.00	1.00	−2.20	1.90	−0.17	1.01	0.17	0.98
Control	−0.00	1.00	−2.08	2.44	−0.21	0.97	0.21	1.01
Judgment of performance	−0.00	1.00	−1.91	2.36	−0.16	0.91	0.16	1.08
Judgment of performance team	−0.00	1.00	−1.69	2.21	−0.25	1.08	0.25	0.86
Communication demand	0.00	1.00	−2.53	1.32	−0.49	1.06	0.49	0.63
Coordination demand	0.00	1.00	−2.08	2.08	−0.26	0.98	0.26	0.97
Team performance monitoring	0.00	1.00	−1.79	2.07	−0.17	0.94	0.16	1.05
Time-share demand	0.00	1.00	−1.32	2.33	−0.01	0.94	0.01	1.08
Team support demand	0.00	1.00	−1.90	2.09	0.04	0.83	−0.04	1.15
Team emotion demand	0.00	1.00	−2.43	1.94	0.05	1.14	−0.05	0.87
Perceived team success	−0.00	1.00	−2.00	1.72	−0.54	0.79	0.50	0.91
CSA	0.00	1.00	−1.36	3.04	0.16	1.14	−0.16	0.83
CSA2	−0.00	1.00	−1.07	2.73	0.23	1.09	−0.23	0.85
Team CSA	0.00	1.00	−0.76	1.92	0.17	1.11	−0.17	0.87
Team CSA2	−0.00	1.00	−1.34	1.75	0.35	0.09	−0.35	0.99
Metacognitive accuracy	−0.00	0.663	−0.79	1.95	−0.08	0.75	0.08	0.56
Metacognitive accuracy2	0.00	0.703	−1.07	1.58	−0.17	0.69	0.17	0.69
Team metacognitive accuracy	0.00	2.168	−4.52	4.55	−0.89	1.15	0.89	2.5
Team metacognitive accuracy2	−0.00	0.800	−1.44	1.35	−0.38	0.46	0.38	0.89

Notes. HRV, Heart rate variability; SAM, self-assessment manikin; TWLQ, Team workload questionnaire; JOP, judgment of performance; RR, R-to-R peak interval; HFHRV, High frequency component heart rate variability; _log, log-transformed; RMSSD, Root mean square of successive RR differences; CSA, Cyber situational awareness; CSA2 and Metacognitive accuracy2, CSA and Metacognitive accuracy without Kill chain scores.

### 3.2 Correlations between HFHRV, SAM, team workload questionnaire, JOP, CSA, and MCA scores

**Figure 4:**
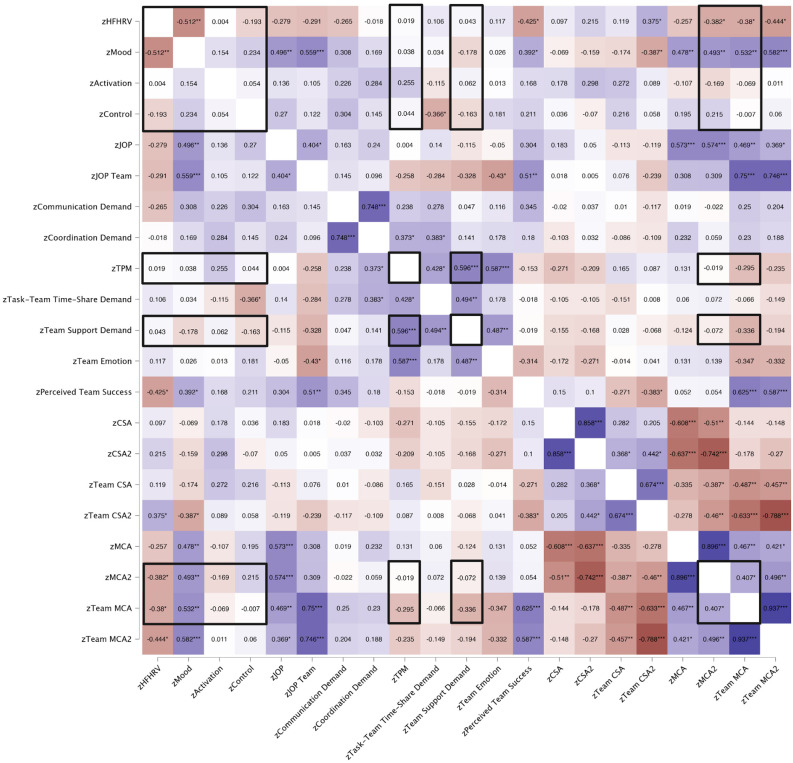
Correlation heat map for HRV, SAM, team workload questionnaire, JOP, CSA, and MCA variables. 2-tailed. *p < 0.050, **p < 0.010, ***p < 0.001. Matrix numbers are Pearson correlation coefficients (*r*) and Spearman’s correlation coefficients (ρ). Pearson’s *r* is indicated with black frames. Red, Negative correlation; Blue, Positive correlation. Color intensity indicates the strength of correlation. HFHRV, High frequency component heart rate variability; JOP, Judgment of performance; TPM, Team performance monitoring; CSA, Cyber situational awareness; MCA, Metacognitive accuracy; CSA2 and MCA2, CSA and MCA without Kill chain scores.

HFHRV was significantly associated with RMSSD (*ρ* = 0.928, *p* < 0.001), indicating that the indices were of good quality. Spearman and Pearson correlations between HFHRV, SAM, team workload questionnaire, JOP, CSA, and MCA variables are presented in Figure 4.

HFHRV was significantly and negatively associated with mood (*p* = 0.003). There were no significant relationships between HFHRV and activation (*p* = 0.841), or control (*p* = 0.457). There were no significant correlations between mood and activation (*p* = 0.602) or control (*p* = 0.382), or between activation and control (*p* = 0.759).

HFHRV was significantly and negatively associated with perceived team success (*p* = 0.017). Mood was significantly and positively associated with perceived team success (*p* = 0.029). Neither HFHRV (*p* = 0.142), mood (*p* = 0.086), activation (*p* = 0.214), nor control (*p* = 0.091) were associated with communication demand. Neither HFHRV, mood, nor activation was associated with any other team workload variables. Control was significantly and negatively associated with time-share demand (*p* = 0.043) but not any other team workload questionnaire items.

HFHRV was not significantly associated with JOP (*p* = 0.122) or JOP team (*p* = 0.106). The mood was significantly and positively associated with JOP (*p* = 0.004) and the JOP team (*p* < 0.001). JOP was not significantly associated with activation (*p* = 0.457), nor control (*p* = 0.135). JOP team was not significantly associated with activation (*p* = 0.567), nor control (*p* = 0.505).

HFHRV was significantly and positively associated with team CSA2 (*p* = 0.035). HFHRV was not significantly associated with CSA (*p* = 0.597), CSA2 (*p* = 0.238), nor team CSA (*p* = 0.516). The mood was significantly and negatively associated with team CSA2 (*p* = 0.029). Mood was not significantly associated with CSA (*p* = 0.706), CSA2 (*p* = 0.384), and nor team CSA (*p* = 0.342). No other significant associations between SAM variables and CSA variables.

Perceived team success was significantly and negatively associated with team CSA2 (*p* = 0.033). No other significant associations between team workload questionnaire scores and CSA scores.

HFHRV was significantly and negatively associated with MCA2 (*p* = 0.031), team MCA (*p* = 0.032), and team MCA2 (*p* = 0.012). HFHRV was not significantly associated with MCA (*p* = 0.156). Mood was significantly and positively associated with MCA (*p* = 0.004), MCA2 (*p* = 0.003), team MCA (*p* < 0.001), and team MCA2 (*p* = 0.004). No other SAM variables were associated with MCA variables. Perceived team success was significantly and positively associated with team MCA (*p* < 0.001) and team MCA2 (*p* < 0.001). No other associations between team workload questionnaire and MCA variables were significant.

Separate linear regression analysis was performed for significant relationships. Table 2 shows the results for the regression analyses.

**Table 2:** Results for linear regression analyses (*N* = 32).

**Predictor**	**Dependent variable**	**β**	***p***	***R^2^_Adj_***	***F*_(1)_**
HFHRV	Mood	−0.512	0.003	0.237	10.644
HFHRV	Perceived team success	−0.382	0.034	0.116	4.949
HFHRV	MCA2	−0.382	0.031	0.117	5.122
HFHRV	Team MCA	−0.380	0.032	0.116	5.049
HFHRV	Team MCA2	−0.441	0.012	0.167	7.223
HFHRV	Team CSA2	0.331	0.064	0.080	3.702
Mood	JOP	0.481	0.005	0.206	9.020
Mood	JOP team	0.518	0.002	0.244	10.999
Mood	Perceived team success	0.384	0.033	0.118	5.018
Mood	MCA	0.424	0.016	0.152	6.575
Mood	MCA2	0.493	0.004	0.217	9.609
Mood	Team MCA	0.532	0.002	0.259	11.858
Mood	Team MCA2	0.569	<0.001	0.302	14.394
Mood	Team CSA2	−0.310	0.085	0.066	3.180
Perceived team success	Team MCA2	0.571	<0.001	0.303	14.058
Perceived team success	Team MCA	0.567	<0.001	0.298	13.760
Perceived team success	Team CSA2	−0.299	0.102	0.058	2.853

Notes. HFHRV, High frequency component heart rate variability; MCA, Metacognitive accuracy; CSA, Cyber situational awareness; JOP, Judgments of performance; CSA2 and MCA2, CSA and MCA without Kill chain scores.

**Figure 5:**
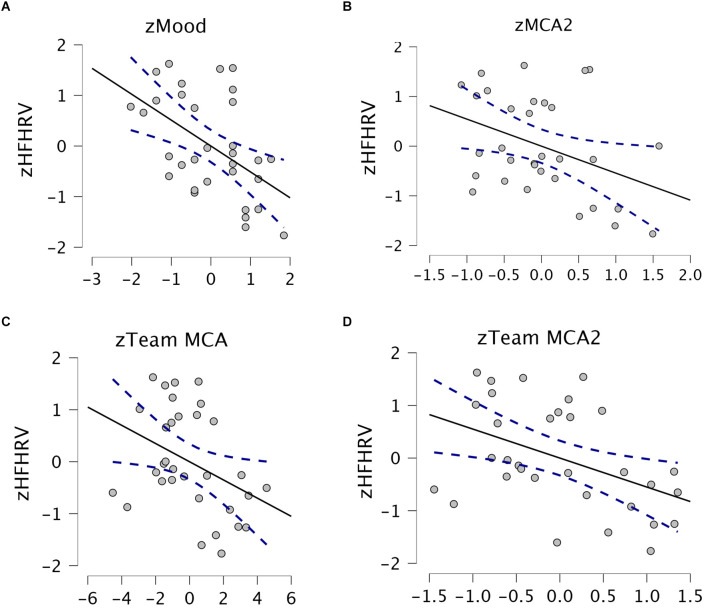
Scatter plots with regression lines. Stapled lines are 95% confidence intervals. **(A)** HFHRV and mood. **(B)** HFHRV and MCA2. **(C)** HFHRV and team MCA. **(D)** HFHRV and team MCA2. HFHRV, High frequency component heart rate variability; MCA, Metacognitive accuracy; MCA2, MCA without Kill chain scores.

HFHRV was a significant negative predictor of mood (*p* = 0.003), perceived team success (*p* = 0.034), MCA2 (*p* = 0.031), team MCA (*p* = 0.032), and team MCA2 (*p* = 0.012). HFHRV was not a significant predictor of team CSA2 (*p* = 0.064). Figure 5 shows regressions for HFHRV and mood, MCA2, team MCA, and team MCA2.

Mood was a significant positive predictor of JOP (*p* = 0.005), JOP team (*p* = 0.002), perceived team success (*p* = 0.033), MCA (*p* = 0.016), MCA2 (*p* = 0.004), team MCA (*p* = 0.002), and team MCA2 (*p* < 0.001). Mood was not a significant negative predictor of team CSA2 (*p* = 0.085).

Perceived team success was a significant positive predictor of team MCA (*p* < 0.001) and team MCA2 (*p* < 0.001). Perceived team success was not a significant predictor of team CSA2 (*p* = 0.102).

### 3.3 Between-group comparisons

Table 3 shows the results from all the comparisons.

**Table 3:** Comparison of differences between groups (*N* = 32).

		**Kruskal-Wallis test**			**Dunn’s *post-hoc***	
**Fixed factors**	**Dependent variables**	***H*(1)**	***p***	**η^2^**	**z**	***p*_bonf_**
Vagal tone groups (low, high)	Communication demand	7.549	0.006	0.218	2.74	0.003
	CSA	0.645	0.422	−0.011	-	
	CSA2	1.484	0.223	0.016	-	-
	Team CSA	0.862	0.353	−0.004	-	-
	Team CSA2	5.207	0.022	0.140	−2.28	0.011
	MCA	1.841	0.175	0.028	-	-
	Team MCA2	6.960	0.008	0.198	2.63	0.004
CSA accuracy (low, high)	MCA	6.937	0.008	0.197	2.63	0.004
Team CSA accuracy (low, high)	Team MCA2	5.205	0.023	0.140	2.28	0.011
		**Pillai’s MANOVA**			**Tukey’s *post-hoc***	
		***F*_(3,28)_**	***p***	**Ω^2^**	**MD**	***p*_bonf_**
Vagal tone groups (low, high)	MCA2	1.975	0.170	0.030	-	-
	Team MCA	6.363	0.017	0.144	1.78	0.017
	Mood	9.026	0.005	0.201	0.94	0.005
		**One-way ANOVA**			**Tukey’s *post-hoc***	
		***F*_(1)_**	***p***	**Ω^2^**	**MD**	***p*_bonf_**
MCA groups (low, high)	HFHRV	4.576	0.041	0.101	−0.71	0.041
Team MCA groups (low, high)	HFHRV	6.301	0.018	0.142	−0.82	0.018
CSA accuracy (low, high)	MCA2	8.393	0.007	0.198	0.67	0.007
Team CSA accuracy (low, high)	Team MCA	14.393	<0.001	0.295	2.55	<0.001

Notes. HFHRV, High frequency component heart rate variability; CSA, Cyber situational awareness. MCA, Metacognitive accuracy; η^2^ and ω^2^, Effect size; CSA2 and MCA2, CSA and MCA without Kill chain scores.

#### 3.3.1 H_1_: Individuals with higher vagal tone have higher metacognitive accuracy and impose lower communication demands on their teams than individuals with lower vagal tone

**Figure 6:**
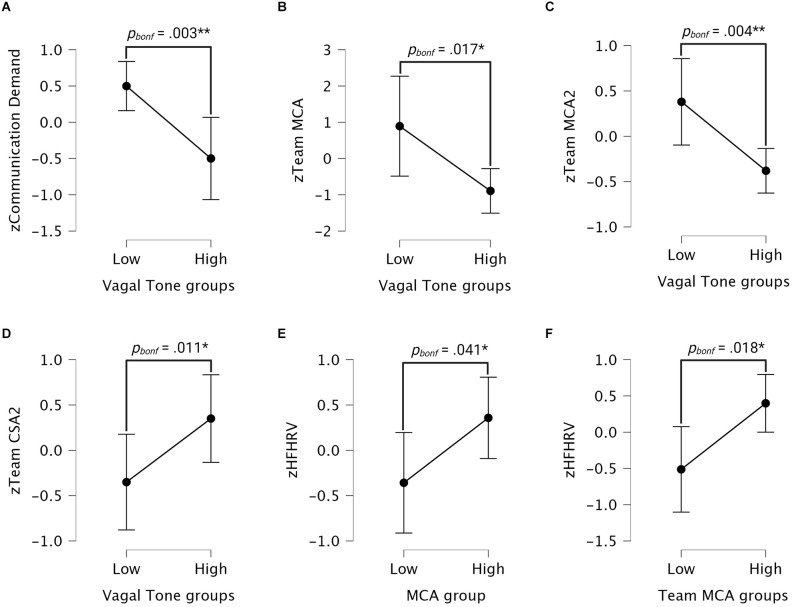
Interval plots for group comparisons. **(A–D)** Interval plots for differences in communication demand, team MCA, team MCA2, and team CSA2 scores between individuals with low and high HFHRV. **(E)** Interval plot showing differences in HFHRV between high and low MCA groups. **(F)** Interval plot showing differences in HFHRV between high and low team MCA groups. Whiskers are 95% confidence intervals. MCA, Metacognitive accuracy; CSA, Cyber situational awareness; HFHRV, High frequency component heart rate variability; CSA2 and MCA2, CSA and MCA without Kill chain scores.

Pillai’s MANOVA was performed using mood, MCA2, and team MCA as dependent variables and vagal tone groups as fixed factor; the Kruskal-Wallis H tests were performed using MCA, team MCA2, CSA, CSA2, team CSA, team CSA2, and communication demand as dependent variables and vagal tone groups as fixed factor. The Pillai test for vagal tone groups was significant (Trace_Pillai_ = 0.269, *F*_(3,28)_ = 5.863, *p* = 0.030). Figures 6A–D shows interval plots for differences between high and low HFHRV groups for MCA, CSA, and communication demand variables.

Communication demand was significantly different between low and high HFHRV groups (*p* = 0.006). Dunn’s *post-hoc* test revealed that individuals with low HFHRV posed significantly more communication demands on their team compared to individuals with high HFHRV (*z* = 2.748, *p_bonf_* = 0.003).

Team MCA was significantly different between low and high HFHRV groups (*p* = 0.017). Tukey’s *post-hoc* test revealed that individuals with low HFHRV had significantly higher team MCA scores than individuals with high HFHRV (MD = 1.78, *p*_bonf_ = 0.017). Team MCA2 was significantly different between low and high HFHRV groups (*p* = 0.008). Dunn’s *post-hoc* test revealed that Individuals with low HFHRV had significantly higher team MCA2 scores compared to individuals with high HFHRV (*z* = 2.63, *p_bonf_* = 0.004). Team CSA2 was significantly different between low and high HFHRV groups (*p* = 0.022). Dunn’s *post-hoc* test revealed that Individuals with low HFHRV had significantly lower team CSA2 scores compared to individuals with high HFHRV (*z* = 2.28, *p_bonf_* = 0.011).

##### 3.3.1.1 Individuals with higher metacognitive accuracy have higher vagal tone than individuals with lower metacognitive accuracy

HFHRV was significantly different between low and high MCA groups (*p* = 0.041). Tukey’s *post-hoc* test showed that individuals with lower metacognitive accuracy had lower HFHRV compared to individuals with higher metacognitive accuracy (MD = −0.71, *p_bonf_* = 0.041). HFHRV was significantly different between low and high team MCA groups (*p* = 0.018). Tukey’s *post-hoc* test showed that individuals with lower team metacognitive accuracy had lower HFHRV compared to individuals with higher team metacognitive accuracy (MD = −0.82, *p_bonf_* = 0.018). Figures 6E,F show interval plots for differences in HFHRV between high and low MCA groups, and high and low team MCA groups, respectively.

#### 3.3.2 H_2_: Individuals with higher vagal tone have different self-reported mood ratings than individuals with lower vagal tone

**Figure 7:**
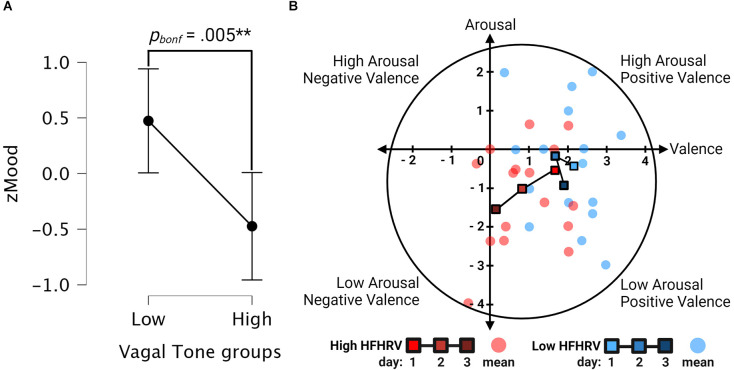
Interval and valence arousal plots. **(A)** Interval plot for differences in mood between high and low HFHRV groups. Whiskers are 95% confidence intervals. **(B)** Valence-arousal plots for high (red) and low (blue) HFHRV groups. Line with squares indicates HFHRV group-means per day. Colors are the brightest for day 1 and darkest for day 3 of the exercise. Transparent circles indicate the mean for all 3 days for each participant.

Results are found in Table 3. Mood was significantly different between low and high HFHRV groups (*p* = 0.005). Tukey’s *post-hoc* test revealed that individuals with low HFHRV had significantly higher mood scores compared to individuals with high HFHRV (MD = 0.94, *p_bonf_* = 0.005). Figures 7A,B show the interval plot for differences in mood between high and low HFHRV groups, and valence-arousal plots for each day for high and low HFHRV groups, respectively. The valence-arousal plots suggested that individuals with high vagal tone had more neutral moods on day 3 of the exercise, while individuals with low vagal tone had more positive moods.

#### 3.3.3 H_3_: Individuals with more correct CSA ratings have higher metacognitive accuracy than individuals with less correct CSA ratings

To assess whether individuals with high MCA were correctly estimating good performance or bad performance, ANOVA and Kruskal-Wallis H tests were performed using MCA2, team MCA, and MCA, and team MCA2 as dependent variables, respectively, and CSA accuracy and team CSA accuracy as fixed factor. Results are found in Table 3. Descriptive statistics for the number and percentage of correct CSA answers on each day for the whole sample, and for MCA groups can be found in Table 4.

**Table 4:** Number and percentage of correct CSA answers for each day (*N* = 32).

			**Low MCA**		**High MCA**		**Low team MCA**		**High team MCA**	
**Variable**	**Count**	**%**	**Count**	**%**	**Count**	**%**	**Count**	**%**	**Count**	**%**
Day 1 Kill chain ratings	4	12.50	2	12.50	2	12.50	2	12.50	2	12.50
Day 1 System critical ratings	6	18.75	2	12.50	4	25.00	2	12.50	4	25.00
Day 1 Severity ratings	2	6.25	0	0.00	2	12.50	0	0.00	2	12.50
Day 1 Action urgency ratings	1	3.12	1	6.25	0	0.00	1	6.25	0	0.00
Day 2 Kill chain ratings	14	43.76	4	25.00	10	62.50	6	37.50	8	50.00
Day 2 System critical ratings	3	9.37	1	6.25	2	12.50	1	6.25	2	12.50
Day 2 Attack severity ratings	7	21.87	2	12.50	5	31.25	2	12.50	5	32.25
Day 2 Action urgency ratings	10	31.25	3	18.75	7	43.75	4	25.00	6	37.50
Day 3 Kill chain ratings	17	53.12	7	43.75	10	62.50	8	50.00	9	56.25
Day 3 System critical ratings	8	25.00	3	18.75	5	31.25	3	18.75	5	31.25
Day 3 Attack severity ratings	7	21.87	1	6.25	6	37.50	3	18.75	4	25.00
Day 3 Action urgency ratings	10	31.25	3	18.75	7	43.75	5	32.25	5	31.25
Day 1 mean team ratings*	13	10.15	5	9.68	8	10.62	5	8.75	8	11.56
Day 2 mean team ratings*	34	26.56	10	24.47	24	28.64	13	22.70	21	30.41
Day 3 mean team ratings*	42	32.81	14	30.72	28	34.89	19	30.31	23	35.31

Notes. CSA, Cyber Situational Awareness; MCA, Metacognitive accuracy. The sum of the percentage of correct sub-group answers is equal to the percentage of correct answers for the whole group multiplied by number of groups. *Count is the total sum of correct CSA assessments across participants’ daily assessments, percentage is the mean of the mean percentage of correct CSA assessments within teams.

MCA was significantly different between CSA accuracy groups (*p* = 0.008). Dunn’s *post-hoc* test revealed that individuals with less accurate CSA ratings had significantly higher MCA scores compared to individuals with more accurate CSA ratings (*z* = 2.63, *p_bonf_* = 0.004). MCA2 was significantly different between CSA accuracy groups (*p* = 0.007). Tukey’s *post-hoc* test revealed that individuals with less accurate CSA ratings had significantly higher MCA2 scores compared to individuals with more accurate CSA ratings (MD = 0.67, *p_bonf_* = 0.007).

Team MCA was significantly different between team CSA accuracy groups (*p* < 0.001). Tukey’s *post-hoc* test showed that individuals with lower team CSA accuracy had higher team MCA scores compared to individuals with higher team CSA accuracy (MD = 2.55, *p_bonf_* < 0.001). Team MCA2 was significantly different between team CSA accuracy groups (*p* = 0.023). Dunn’s *post-hoc* test showed that individuals with lower team CSA accuracy had higher team MCA2 scores compared to individuals with higher team CSA accuracy (*z* = 2.28, *p_bonf_* = 0.011).

## 4 Discussion

In this study, we aimed to assess some of the neurocognitive assumptions of the OLB model (Knox et al., 2018) to begin validating its potential as a neuroergonomic approach for CSA communication in cyber threat situations. This was done in a sample of cyber cadets participating in a cyber engineering exercise by using a combination of psychophysiological, CSA, metacognitive, and team measurements targeted at assessing some of the OLB model’s implicit underlying neurocognitive assumptions (Jøsok et al., 2016; Knox et al., 2018). The OLB model outlines an adaptive three-step metacognitive control process for how to communicate efficiently between individuals under varying cyber situational dynamics (Knox et al., 2018). In the OLB model, the communicator integrates self-referential, self-other, situational, and task-goal information to ground communication and establish a shared CSA. This requires the prefrontal part of the brain to coordinate activity across brain structures and networks (Nee and D’Esposito, 2016; Morales et al., 2018; Nee, 2021; Friedman and Robbins, 2022).

Being a proxy for activity in prefrontal structures relevant for OLB-ing (Brunoni et al., 2013a; Nikolin et al., 2017; Chand et al., 2020), we hypothesized that individuals with high vagal tone had higher metacognitive accuracy and imposed less communication demands on their teams compared to individuals with low vagal tone (H_1_). In our initial analyses, we found that vagal tone was associated with higher metacognitive accuracy for prospective judgments about individual performance and team performance. This is in line with previous studies suggesting that the DLPFC is involved in prospective metacognitive performance estimations (Vaccaro and Fleming, 2018; Fleur et al., 2021). In our between-group analyses comparing differences in metacognitive accuracy and communication demands between individuals with low and high vagal tone, we found that team-level metacognitive accuracy was significantly higher in the high vagal tone groups. Individual-level metacognitive accuracy was not significantly different between vagal tone groups. That the findings regarding individual metacognitive accuracy for the whole sample could not be replicated in the sub-group analysis could be due to the size of the sub-groups. Both vagal tone (Brunoni et al., 2013a; Nikolin et al., 2017; Chand et al., 2020) and prospective metacognitive judgments are influenced by activity in the DLPFC (Fleur et al., 2021). Vagal tone is, however, influenced by several physiological processes other than DLPFC activity (Task Force of the European Society of Cardiology and the North American Society of Pacing and Electrophysiology, 1996), as opposed to prospective metacognitive judgments which are dependent on the DLPFC (Vaccaro and Fleming, 2018). Thus, to make sure to account for this possible influence on our results, we did a follow-up analysis assessing differences in vagal tone between individuals with high and low individual and team-level metacognitive accuracy. Vagal tone was higher in individuals with higher metacognitive accuracy for both individual and team-level performance estimations. Finally, in line with our hypothesis, we also found that individuals with high vagal tone imposed lower communication demands on their team compared to individuals with low vagal tone.

Our findings should be interpreted in light of previous research suggesting that communication inefficiencies are one of the main problems facing SOC teams (Agyepong et al., 2019; Ask et al., 2021a). These inefficiencies occur both between analyst-level personnel, for example where cyber operators fail to communicate threat and defense knowledge, ultimately resulting in team members wasting time researching a problem that someone on the team has already solved (Jariwala et al., 2012; Ahrend et al., 2016; Skopik et al., 2016; Staheli et al., 2016). Communication inefficiencies also occur between analysts-level and decision-making personnel, where critical information for establishing CSA may get lost as it is communicated from technical personnel and upwards in the decision-making hierarchy to less technical personnel (Staheli et al., 2016; Jøsok et al., 2017; Knox et al., 2018). Suggestions for improving communication between SOC teams have been proposed, such as establishing shared mental models for communication and transactive memories about the expertise and knowledge of team members (Steinke et al., 2015; Hámornik and Krasznay, 2018). This is considered especially critical during cyber threat situations where time pressure is high, and one-way communication is required where all critical information is communicated at once. Our findings may indicate that OLB-related metacognitive processes may help facilitate and proliferate a better understanding of the knowledge and competencies of individuals on the team, and that this is related to establishing better team-level CSA.

Our sample consisted of cyber cadets that know and are used to interact with each other. Nevertheless, our findings may also have relevance for the challenges that arise when information has to be communicated between people that have different priorities spanning the cyber-physical and strategic-tactical dimensions of cyber operations (Jøsok et al., 2016, 2017). Suggested approaches for developing shared mental models for SOC team communication mainly focus on establishing what information should be communicated ahead of time, or understanding the procedures related to performing the tasks of different members of the team (Steinke et al., 2015; Hámornik and Krasznay, 2018). Due to different stakeholders having different communication needs (Ahrend et al., 2016; Jøsok et al., 2016), it is hard to define any set protocol for what to communicate, and it is generally understood that approaches must be adapted to the needs of SOC teams and their clients (Ask et al., 2021a). This is especially the case for communication between technical and non-technical personnel in situations where communication must be adapted according to changing situational dynamics (Jøsok et al., 2017; Ask et al., 2021a). Thus, developing good process-based models for dynamic communication that can be implemented in cyber defense training and education is urgently needed (Knox et al., 2018; Ask et al., 2021a). This is where the findings in the present study may be relevant. Due to the cognitive load associated with the cybersecurity working environment, models for communication must be feasible to apply in high-stress situations to be sustainable. Applying metacognitive processes in a three-step fashion as outlined in the OLB model may also provide opportunities for regulating stress prior to communication. This may result in an increased capacity for processing the information that is communicated, or remembering what should be communicated. Thus, while having a strategic plan for what to communicate during a cyber threat situation is considered crucial for communication to be efficient (Steinke et al., 2015; Hámornik and Krasznay, 2018), approaches such as the OLB model (Knox et al., 2018) that are designed for situational adaptation may serve as a neuroergonomic complement to facilitate strategic communication.

The present findings may shed light on results from other studies on cyber defense teams indicating that experts impose less communication demands on their teams than novices (Buchler et al., 2016; Lugo et al., 2017a). This may suggest that experts have a better metacognitive understanding of team competence and more efficient (shared) mental models for communication than novices. If expert team communication efficiency reflects a higher rate of adoption of neuroergonomic strategies in response to working under stress, then even unconscious strategy adoption may result in a higher number of shared, albeit implicit mental models for team communication. Overlap of implicit mental models may depend on the degree of strategy convergence that is enforced by environmental pressure. Learning cognitive skills is associated with reduced activity in brain areas responsible for the skill, which is considered a marker for increased processing efficiency (Fleur et al., 2021). However, having an accurate mental model of the competencies of team members may serve as an anchor for cognitive effort one considers necessary for performing well during a cyber threat situation. An example may be finding a compromise between social loafing and effort where the highest level of cognitive output can be sustained for the longest period of time. Previous research has indicated that level 3 situational awareness is more taxing on working memory than preceding levels (Gutzwiller and Clegg, 2013). Cognitive strategies that serve to balance personal knowledge acquisition with cognitive offloading without compromising team performance may compete with strategies for maximizing expertise and being the one to solve any given problem. At intermediate levels of environmental stress, any one strategy, or a flexible combination of the two, maybe equally sustainable. Under increasing loads, however, strategies should converge on those optimizing for balancing cognitive offloading with sustained effort, which may favor metacognitive team processes over individual processes. Strategy convergence may, however, depend on how salient tasks are, and the interests and priorities of the individual (Wickens et al., 2015).

It has been suggested that individual and team-based metacognitions depend on different processes (Shea et al., 2014). Interestingly, while being associated with the accuracy of prospective performance judgments, vagal tone was not associated with prospective judgments of confidence in individual or team performance, but was negatively associated with retrospective confidence judgments of team success. In turn, retrospective judgments of performance were negatively associated with the accuracy of prospective metacognitive judgments of team performance, but not individual performance. Retrospective judgments are associated with activity in the parahippocampal structures and the inferior frontal gyrus (Vaccaro and Fleming, 2018), but are typically assessed at the individual level in neurocognitive studies and not in a team setting. While fast acting connections between the DLPFC and hippocampal structures have been established (Friedman and Robbins, 2022), recent studies show that tracking dynamic social behavior is dependent on interactions between the DLPFC and dorsomedial PFC (McDonald et al., 2020).

This article argues that the processes outlined by the OLB model rely on the coordinated and flexible transition between FPN- and DMN-related information processing, which is a cognitive control process (Nee and D’Esposito, 2016; Nee, 2021; Friedman and Robbins, 2022). Vagal tone measured at rest is associated with connectivity between the DMN and FPN (Chand et al., 2020). The transition between cognitive processes can either be subject to self-regulated metacognitive control (Shimamura, 2008) or stimulus-driven as a result of stress and emotional influence (Baek and Falk, 2018; Poth, 2021). A recent study found that the FPN was involved in metacognitive judgments along with DMN structures, where activity in both the DLPFC and MPFC was negatively associated with confidence judgments (Morales et al., 2018). Emotions are processed differently by DMN and FPN structures (Golkar et al., 2012; Terasawa et al., 2013; Fujimoto et al., 2021; Nejati et al., 2021), while stress disrupts connectivity between the FPN and DMN along with its association with vagal tone (Chand et al., 2020). Previous studies on cyber cadets identified several emotional and self-regulatory processes that may have differing effects on teamwork and communication (Lugo et al., 2016, 2017b, 2021; Knox et al., 2017; Jøsok et al., 2019; Ask et al., 2021b). As the DLPFC is involved in mood processing (Golkar et al., 2012; Nejati et al., 2021), we hypothesized that individuals with higher vagal tone had different self-reported mood ratings than individuals with lower vagal tone (H_2_). In line with our second hypothesis, we found that vagal tone was negatively associated with mood. This finding was also replicated in our subgroup analysis where individuals with higher vagal tone had lower self-reported mood than individuals with lower vagal tone.

The valence-arousal plots showing daily mood and arousal for individuals with high and low vagal tone indicated that individuals with high vagal tone had more neutral moods on day 3 of the exercise, while individuals with low vagal tone had more positive moods. In a previous study, we found that variations in daily affect were associated with experienced team workloads among cyber cadets participating in a cyber defense exercise (Ask et al., 2021b). While the significance of such findings may be unclear with respect to exercise outcomes (Lund, 2022), the findings in the present study may serve to further elucidate their relevance beyond suggesting that individual characteristics influence team dynamics. While stress and urgency may disturb analytic cognitive processes (Poth, 2021), positive moods, as opposed to neutral moods may also result in transitioning from analytical processing to stimulus-oriented processing (Baek and Falk, 2018), for example as a result of optimism bias and mood congruent processing, lack of suspicion, or overconfidence (Vishwanath et al., 2018; Canham et al., 2022; Sütterlin et al., 2022). In practice, this may result in reduced situational understanding, as indicated in our study by positive moods being a significant negative predictor of both individual and team-level prospective metacognitive judgments of performance, as well as being a positive predictor of retrospective judgments of team success. Retrospective judgments of team success were as noted negatively associated with the accuracy of team-level metacognitive judgments. These findings also mirror other studies where overconfidence has been associated with worse threat detection abilities among IT and cybersecurity personnel (Butavicius et al., 2016; Jampen et al., 2020; Sütterlin et al., 2022).

In the present study, having high metacognitive accuracy could be either due to accurately judging performance as bad or as good. Thus, it was technically possible that individuals with high metacognitive accuracy could perform equal to- or even worse on CSA estimations than individuals with low metacognitive accuracy as long as they were more correct in their performance estimations. Because good cyber defense decision-making is based on having accurate CSA (Barford et al., 2009), and metacognitive accuracy is necessary for correct SA (Endsley, 2020), we also tested the hypothesis that individuals with higher metacognitive accuracy would have more correct CSA ratings than individuals with lower metacognitive accuracy (H_3_). We found that individuals with higher metacognitive accuracy also had more correct CSA ratings at both the individual and team-level. This supports our hypothesis and the findings of Endsley (2020). No measurement of team dynamics other than retrospective judgments of performance was associated with accurately judging team performance and team-level CSA ratings. This stresses the importance of training metacognitive skills to ensure that SOC teams are able to generate and share accurate CSA. In other words, team based training in absence of metacognitive training may not efficiently provide all the skills necessary for ensuring SOC team performance. This should arguably occur during education rather than relying on individual SOC teams to ensure that new recruits learn metacognitive skills. However, this may challenge traditional educational and organizational practices (Jøsok et al., 2017; Knox et al., 2018) as reflected in the plethora of challenges SOC teams face (Agyepong et al., 2019; Ask et al., 2021a). Originally developed as a pedagogical tool, the OLB model (Knox et al., 2018) may serve as a flexible and cost-effective approach to metacognitive training that is easy to implement across learning situations and institutions.

To the best of our knowledge, this is the first study providing neuroergonomic insights into the relationship between communication in teams and metacognitive CSA accuracy in a cybersecurity setting. While we aimed to provide neuroergonomic support for the OLB model, the present findings could also be used to argue for the importance of psychophysiological measurements in recruitment, training, and performance monitoring. Previous research found associations between vagal tone and performance among tactical personnel in non-cybersecurity settings (including military; Tomes et al., 2020). The present study is the first to show that vagal tone may also serve as an indicator of performance in a cybersecurity setting. To the extent that vagal tone reflects the ability for self-regulation (e.g., Segerstrom and Nes, 2007; Reynard et al., 2011), our findings are an addition to a growing body of literature showing relationships between self-regulation and movements in the Hybrid Space (Knox et al., 2017, 2019; Jøsok et al., 2019). Finally, the present findings also provide support for the scarce literature on relationships between vagal tone and metacognitive accuracy (Meessen et al., 2018).

### 4.1 Limitations and future directions

The aim of this study was to assess the neurocognitive assumptions of the OLB model (Knox et al., 2018) to determine its potential as a neuroergonomic approach to improve communication. We did this using vagal tone as a proxy for neural activity thought to be relevant for OLB execution (Brunoni et al., 2013a; Nikolin et al., 2017; Chand et al., 2020). The present study goes some length in achieving this, however, future studies should use an intervention design where some participants are trained in explicitly applying the model. While the vagal tone is considered a stable trait that is hard to change with intervention (e.g., Brunoni et al., 2013b; Wheeler et al., 2014; Neyer et al., 2021), metacognition is something that can be trained (Jøsok et al., 2016; Fleur et al., 2021). Thus, it would be both interesting and necessary to assess whether individuals who are trained in applying the OLB model but have low vagal tones perform better than individuals who are not trained in using the model but have high vagal tones. To ensure that this is done in a naturalistic setting may require experimental collaboration between cognitive scientists and cyber defense exercise organizers (Ask et al., 2021a).

Albeit comparing team-level and individual level metacognitive accuracy is not addressed in this study, Table 4 indicates that the number of correct individual answers for each CSA item per day is mostly overlapping between individuals with high individual- and team-level metacognitive accuracy, although slightly favoring individual metacognitive accuracy. However, when looking at the descriptive statistics for the mean percent of correct answers within teams, the proportion of the mean of correct team answers appears larger for individuals with high team metacognitive accuracy, even though the number of their individual contributions is lower. As noted in previous studies (Ask et al., 2021a, b), including both team- and individual-level measurements is important to develop SOC team performance metrics, and future studies should assess how team-level and individual-level metacognition contributes to team performance. In Table 4 it appears that individuals with high individual- and team-level metacognitive accuracy on day one were completely overlapping. The OLB model suggests using metacognition to make communication of CSA between team members more efficient (Knox et al., 2018). Supra-individual metacognitive processes are suggested to be involved in inter-individual cognitive control (Shea et al., 2014), thus it would be interesting to see whether individuals with high metacognitive accuracy in the beginning of a cyber defense exercise influence the evolution of team performance.

As part of the exercise, the cadets were also assessed on leadership skills and factors other than CSA and mission success. It is possible that some participants included these factors when making prospective judgments of their own and the team performance, thus inflating or deflating their confidence relative to our outcome measurements. Because excluding this possibility would require probing each participant about what they based their estimations on, it is safer to assume that our metacognitive accuracy estimates are conservative. Furthermore, there have been reported sex differences in relationships between social orientations and vagal tone (Lischke et al., 2018). Due to conducting the study in a security setting, we did not ask participants to provide information on their sexes. While the sex of participants is commonly underreported in cybersecurity studies (Ask et al., 2021a), a recent study suggested that sex may play a role in communication among cyber engineers (Fisher, 2022). Future studies should therefore make an effort to also assess whether findings are differentially influenced by sex. A final limitation of the current study is the sample size. While the present study included the entire cohort of the studied population, more studies are needed to replicate findings.

## 5 Conclusion

Prefrontally modulated vagal tone, an indicator of activity in brain structures relevant for coordinating the cognitive processes underlying OLB model execution, is associated with metacognitive cyber situational awareness and imposing lower communication demands on the team. Based on the assumption that individuals working in high-stress-, high-cognitive load-environments will choose neuroergonomic cognitive strategies to reach task goals, the present findings suggest that the OLB model is neuroergonomic in such environments. Individuals with higher vagal tone had more neutral moods which could be necessary for allocating more attentional resources to analytical processing. Furthermore, individuals with higher CSA had higher metacognitive accuracy compared to individuals with lower CSA supporting previous studies suggesting that metacognitive accuracy is necessary for achieving situational awareness. The present study highlights the potential of using neurophysiological measurements as performance indicators. Future studies are needed to explicitly address the effect of using the OLB model as the basis for a metacognitive intervention to improve communication and team performance, as well as replicating the findings of the present study.

## Data Availability Statement

The datasets presented in this article are not readily available because access to raw and processed data is restricted in accordance with agreement between the researchers and the Norwegian Defense University College, Cyber Academy (NDCA). Requests to access the datasets should be directed to corresponding author.

## Ethics Statement

Ethical review and approval was not required for the study on human participants in accordance with the local legislation and institutional requirements. The patients/participants provided their written informed consent to participate in this study. The present study conformed to institutional guidelines and was eligible for automatic approval by the Norwegian Social Science Data Services’ (NSD) ethical guidelines for experimental studies. Participation was voluntary and all participants were informed about the aims of the study, the methods applied, that they could withdraw from participation at any time and without any consequences, and that if they did so all the data that was gathered from them would be deleted. After volunteering to participate in the study, participants were asked to provide informed consent on the first page of an online form where baseline data was collected. Participants were asked to generate and remember a unique participant ID that they would use during data collection for the duration of the study.

## Author Contributions

TA: study design and methods, data collection, analysis, writing of original draft, review and editing. BK: study design, writing of original draft, review and editing. RL and SS: study design and methods, review and editing. IH: exercise organization, scoring of CSA questionnaires for analysis, writing of original draft. All authors contributed to the article and approved the submitted version.
